# Accelerated cloud and GPU-based simulations for quantification of relaxation times: an example with MOLLI

**DOI:** 10.1186/1532-429X-18-S1-P42

**Published:** 2016-01-27

**Authors:** Georgios Kantasis, Christos G Xanthis, Anthony H Aletras

**Affiliations:** 1 Department of Computer Science and Biomedical Informatics, University of Thessaly, Lamia, Greece; 2 Department of Medicine, Aristotle University of Thessaloniki, Thessaloniki, Greece; 3 Department of Clinical Physiology, Lund University, Lund, Sweden

## Background

Quantification of native T1 in the myocardium has the potential to become an important biomarker for assessing specific cardiomyopathies. However, the accuracy and precision of the clinically available CMR mapping techniques are affected by several parameters [1].

A recently developed method that allows for accuracy improvement of MOLLI quantification was presented [2, 3]. This method is based on comprehensive, GPU-based MR simulations of the identical pulse sequence on a large population of spins resulting in a database of all possible outcomes. MR simulations are computationally intensive with long execution times not well suited for clinical applications. The aim of this study was to improve performance to more clinically acceptable levels. We hypothesized that this could be accomplished by a cloud and GPU-based implementation approach.

## Methods

An Amazon Web Services [4] cloud-based cluster consisting of g2.2 × large computer instances was utilized. A variable number of instances were used as slave nodes. Each node performed the simulation of the entire pulse sequence on a subset of the spin population and one of them was also assigned the role of job manager (master node). The resulting database entries were then collected from the slave nodes and joined together on the master node.

The simulated pulse sequence was a MOLLI with a 5(3p)3 acquisition scheme consisting of 156877 discrete time-steps. The spin population consisted of 2756061 spins with variable T1 and T2 combinations, covering physiological myocardial and blood relaxation times.

The execution times were measured and the total speedup was calculated for 1 to 16 nodes. The total speedup was calculated according to the total execution time and the GPU speedup according to solely the execution time of the GPU part of the simulation. The overhead, defined as the time of data transfers, joining the database and preparing to run the simulation, was also measured.

## Results

**Figure 1:**
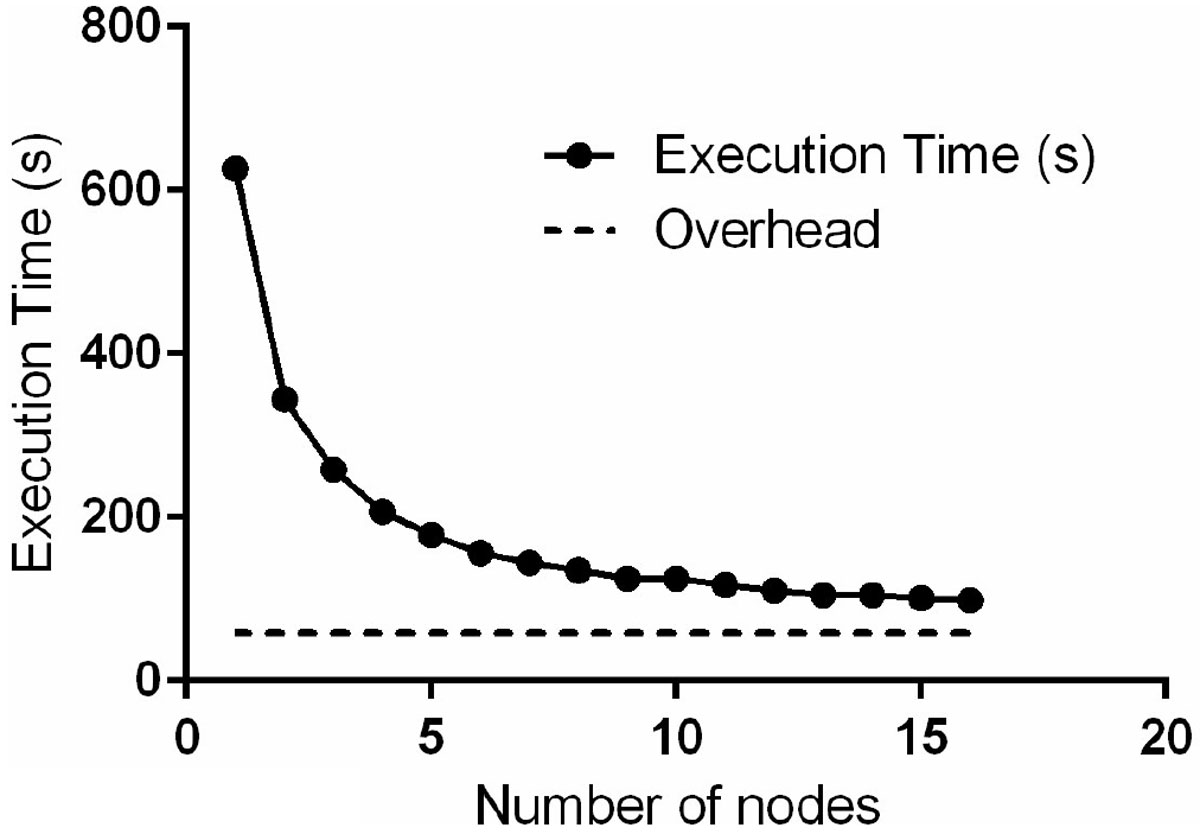
**Total execution times**. The circled line is the total execution time. The dashed line is the measured overhead.

**Figure 2:**
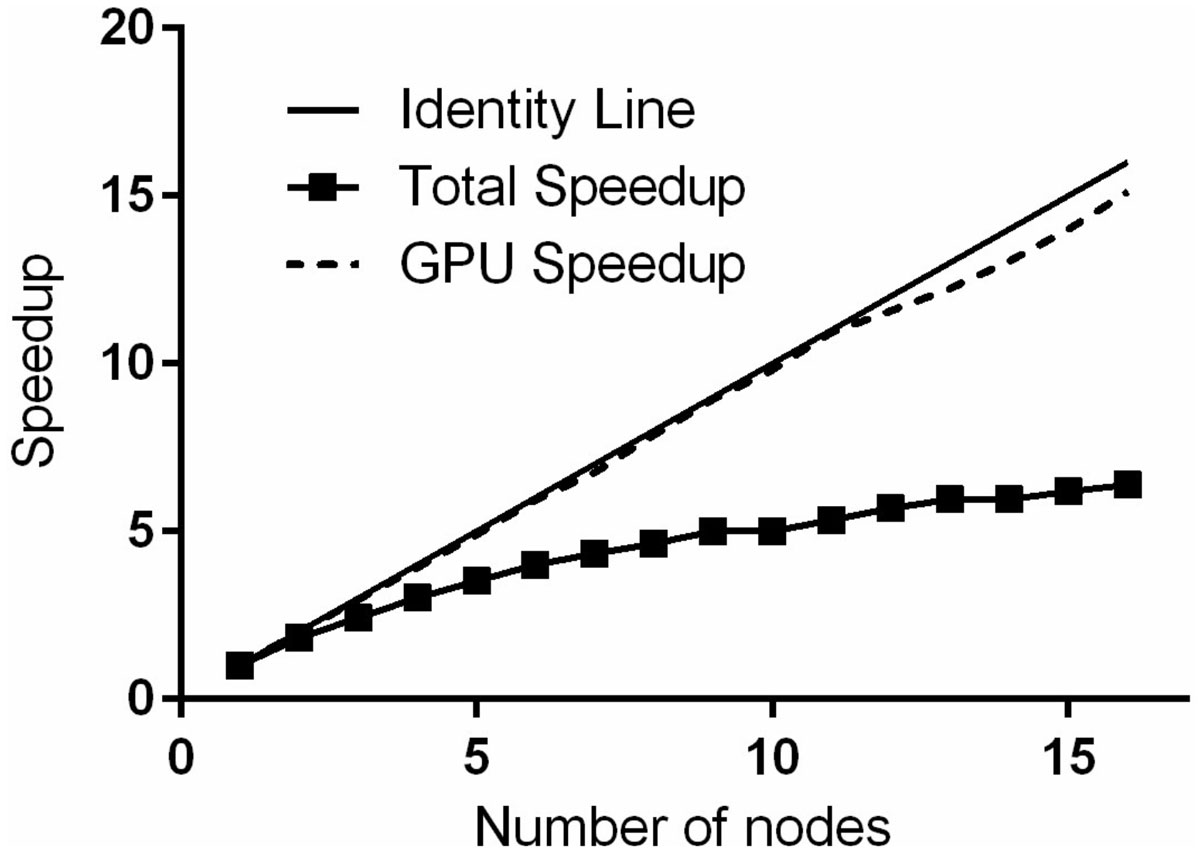
**Effective speedup of the cluster**. The squared line represents the total speedup. The dashed line represents the GPU speedup. The solid line is the identity line.

Figure 1 shows the total execution times versus the number of nodes. The execution times measured ranged from 626 sec (single node) to 98 sec (16 nodes). The overhead was found to be 58 sec. In Figure 2, the squared line shows the total speedup achieved on the cloud versus the number of nodes utilized whereas the dashed line shows the corresponding GPU usage speedup. For the 16-node setup, the total experiment demonstrated a speedup of 6.39 while the GPU usage demonstrated a speedup of 15.13.

## Conclusions

In this study, a cloud-based approach of simulation-based corrections on MOLLI T1 mapping data [2, 3] was used. The measured speedups reflect the benefits of distributing the computational problem across multiple nodes within the cloud. This work suggests that, in the future, using the cloud may allow for simulation-based correction of patient data to become useful in the clinic.
